# Reduction of *Salmonella* Shedding by Sows during Gestation in Relation to Its Fecal Microbiome

**DOI:** 10.3389/fmicb.2017.02219

**Published:** 2017-11-10

**Authors:** Guillaume Larivière-Gauthier, Alexandre Thibodeau, Ann Letellier, Étienne Yergeau, Philippe Fravalo

**Affiliations:** ^1^NSERC Industrial Research Chair in Meat-Safety (CRSV), Faculty of Veterinary Medicine, University of Montreal, Saint-Hyacinthe, QC, Canada; ^2^Centre INRS-Institut Armand-Frappier, Institut National de la Recherche Scientifique, Université du Québec, Laval, QC, Canada

**Keywords:** *Salmonella*, sows, fecal microbiota, gestation, excretion

## Abstract

Pork meat is estimated to be responsible for 10–20% of human salmonellosis cases in Europe. Control strategies at the farm could reduce contamination at the slaughterhouse. One of the targeted sectors of production is maternity, where sows could be *Salmonella* reservoirs. The aim of this study was to assess the dynamics of shedding of *Salmonella* in terms of variation in both shedding prevalence and strains excreted during gestation in Quebec’s maternity sector. The evolution of the fecal microbiota of these sows during gestation was also assessed to detect bacterial populations associated with these variations. A total of 73 sows both at the beginning and the end of the gestation were randomly selected and their fecal matter was analyzed. *Salmonella* detection was conducted using a method that includes two selective enrichment media (MSRV and TBG). Nine isolates per positive samples were collected. Among the 73 sows tested, 27 were shedding *Salmonella*. Sows in the first third of their gestation shed *Salmonella* significantly more frequently (21/27) than those in the last third (6/46) (χ^2^ *P* < 0.05). The shedding status of 19 of the sows that were previously sampled in the first third of their gestation was followed, this time in the last third of their gestation, which confirmed reduction of shedding. Using 16S rRNA gene sequencing and qPCR, significant differences between the fecal flora of sows at the beginning and the end of the gestation, shedding *Salmonella* or not and with different parity number were detected. Using MaAsLin, multiple OTUs were found to be associated with the time of gestation, the status of *Salmonella* excretion and parity number. Some of the identified taxa could be linked to the reduction of the shedding of *Salmonella* at the end of gestation. In this study, we showed that the level of *Salmonella* shedding was variable during gestation with significantly higher shedding at the beginning rather than at the end of gestation. We also observed for the first time a significant change in the microbiota during sow gestation and identified interesting taxa which could be linked to a reduced *Salmonella* shedding.

## Introduction

In Canada, *Salmonella enterica* is estimated to cause 269.26 infections per 100,000 inhabitants each year, confirming this pathogen as a public health priority (Thomas et al., 2013). Contamination in humans causes gastroenteritis and occurs from the consumption or mishandling of contaminated meats. The economic impact of productivity losses and medical care expenses caused by salmonellosis is estimated to reach 3.3 billion dollars in the US alone each year (Hoffmann et al., 2012). In Canada, the part of salmonellosis that are caused by consumption of pork products is not known. However, in Europe, it has been estimated that between 10 and 20% of all salmonellosis cases are due to the consumption of contaminated pork meat (EFSA Panel on Biological Hazards [BIOHAZ], 2010).

*Salmonella* can contaminate swine on the farm and most of the serotypes can be carried asymptomatically in their intestinal tract, gut-associated lymphoid tissue and tonsils, and enter slaughterhouses with the animals where it can contaminate the meat (Boyen et al., 2008). *Salmonella* can enter the farm through multiple pathways, such as contaminated feeds or employees (Funk and Gebreyes, 2004). One of the most important sources of new strains on a farm is the introduction of already contaminated animals (Funk and Gebreyes, 2004; Gotter et al., 2012). Once on the farm it can be very difficult to eliminate using regular washing and disinfection methods and residual strains can contaminate newly arrived *Salmonella* free animals (Argüello et al., 2011; Dewaele et al., 2012). In certain parts of Canada up to 60% of the swine farms are contaminated (Rajic et al., 2005; Farzan et al., 2008) with some studies showing 25% of contaminated animals (Wilkins et al., 2010). Since it has been shown that the entrance of contaminated pigs into slaughterhouses is linked with an increased risk for the contamination of carcasses, a reduction at the first stages of the production could be an important step in the reduction of the contamination of the meat (Letellier et al., 2009; EFSA Panel on Biological Hazards [BIOHAZ], 2010).

Canadian sows in the provinces of Saskatchewan and Alberta have been shown to be highly contaminated by *Salmonella*, with levels of shedding higher than swine in fattening at 38% compared to 25% respectively (Wilkins et al., 2010). These contaminated sows are believed to be important reservoirs of contamination during the maternity phase where *Salmonella* could be transmitted to the piglets, spreading contamination to later stages of production (Belœil et al., 2004; EFSA Panel on Biological Hazards [BIOHAZ], 2010; Hill et al., 2015). Variation in the percentage of sows excreting *Salmonella* during the maternity production cycle has already been observed (Nollet et al., 2005b; Magistrali et al., 2011). However, information is still scarce on the dynamics of shedding by sows during gestation, on the real impact of contamination on the rest of production and the factors that are responsible for this variation.

Sequencing technologies have helped to show the importance of microbiota in fighting the colonization of possibly harmful bacteria by competitive exclusion, stimulation of immunity and the production of antimicrobial substances. In the case of *Salmonella*, it has been shown that short chain fatty acids (SCFA, e.g., butyric acid), that are by-products of the microbiota’s digestion of complex sugar, can reduce virulence of *Salmonella* by lowering the activation of the *Salmonella* Pathogenicity Island-1 (SPI-1) (Gantois et al., 2006). Studies have also shown that swine excreting *Salmonella* had a different microbiota than swine that were not excreting (Fravalo et al., 2013; Lebel et al., 2016). Similarly, it has been shown that swine with different levels of *Salmonella* shedding after an experimental infection had different microbiota compositions (Bearson et al., 2013). Interestingly, in other species, fecal microbiota has been shown to vary during gestation (Collado et al., 2008; Koren et al., 2012). However, this variation, and its possible impact on the shedding of *Salmonella*, has not been studied in sows.

The aim of this study was to assess the dynamics of *Salmonella* shedding by sows in terms of variation in both shedding prevalence and strains excreted during gestation in an industrial setting in Quebec. The evolution of the fecal microbiota of these sows during gestation was also assessed in order to detect bacterial populations that could be associated with the observed variations.

## Materials and Methods

### Sampling

All animal experimentations were approved by the ethics committee of the Faculty of Veterinary Medicine of the University of Montreal, certificate number 14-Rech-1714. The protocol was approved by the ethics committee of the Faculty of Veterinary Medicine of the University of Montreal. A total of 73 sows at various gestation stages and parity (between 1 and 8; **Table 1**) were randomly selected from a breeding farm known for its frequent *Salmonella* contamination. All sows were fed with the same feed during all the gestation. For each selected sow, 100 g of fresh fecal matter was collected and analyzed. After the first sampling, 19 out of the 73 sows that were sampled at the beginning of their gestation (first 50 days) were sampled a second time at the end of this period (last 50 days). For each sampled sow, 1 g of feces was subsampled in the farm and immediately frozen in liquid nitrogen for 16S rRNA gene amplicon MiSeq sequencing.

**Table 1:** Number of sows sampled in each parity and their *Salmonella* shedding status.

Parity	1	2	3	4	5	6	7	8
Number of sows	21	8	11	8	10	7	5	3
% of excreting sows	29	50	36	25	40	57	20	67


### Detection

*Salmonella* detection was conducted using a method adapted from the method described by De Busser et al. (2013). The method was optimized by collecting isolates from multiple selective enrichment media as well as from two migration distances on MSRV to obtain the best possible description of the eventual variety of strains contained in the samples. First the samples (100 g) were pre-enriched in buffered peptone water (BPW) (1:10 w/v, 18 h, 37°C) (Biokar diagnostic, Beauvais, France). Modified Semi-Solid Rappaport-Vassiliadis Agar (MSRV) (24–48 h, 42°C) (Lab M, Heywood, United Kingdom) and Tetrathionate Brilliant Green Bile Broth (TBG) (24 h, 42°C) (BD Difco, Franklin Lakes, NJ, United States) selective enrichment media were then used in parallel and further inoculated on Brilliant Green Sulfa Agar (BGS) (BD Difco, Franklin Lakes, NJ, United States). Two BGS plates were inoculated from the MSRV from two different regions of the migration zone (far from and near the inoculation spot) and one from the TBG. A maximum of nine (3 per BGS plate) isolates per positive samples were collected. Suspect colonies on BGS were confirmed as *Salmonella* using triple sugar iron agar slants (Lab M, Heywood, United Kingdom) and urea agar slants (Lab M, Heywood, United Kingdom) followed by seroagglutination using *Salmonella* O antiserum Poly A-I + Vi (Statens Serum Institute, Denmark).

After isolation, the motility of the isolates originating from samples that were only positive for TBG and from samples where isolates from multiple genotypes were detected with MRSV was confirmed. Each isolate was grown individually for 24 h at 37°C in 10 ml of BPW. Then 100 μl of the enrichment broth was inoculated on the middle of an MSRV plate and incubated for 24 h at 42°C before the migration zone was observed. Each of the isolate was tested in triplicate.

Percentages of *Salmonella* positive animals at the beginning and the end of gestation were compared using Fisher’s exact test for the first random sampling and McNemar’s test for the second sampling where each sow was sampled twice (GraphPad v6, Prism, LaJolla, CA, United States). The link between shedding and the parity of sows was also assessed using a logistic regression (R 3.4.1).

### Genotypic Characterization

Each isolate was genotyped using a high resolution melt (HRM) technique adapted from the method described by Bratchikov and Mauricas (2011). The precise melting curves of three genomic regions, two CRISPRs (*cr1* and *cr2*) and one VNTR (*yohm*), were analyzed after PCR amplification using a LightCycler 96 real time PCR (Roche diagnostics, Mannheim, Germany). The combined analysis of these three curves was associated with an HRM type. A variation in the melting curve profile from one of these three regions was considered to reveal a new HRM type. Representative isolates of each of the different types were serotyped by the veterinary epidemiosurveillance laboratory of the Ministère de l’Agriculture, des Pêcheries et de l’Alimentation du Québec.

### 16S rRNA Gene Sequencing

Total bacterial DNA was extracted using mechanical and chemical lysis followed by a phenol/chloroform purification. Five hundred μg of the frozen fecal content was added to 500 μl of lysis buffer [Tris-HCl 500 mM pH 8, EDTA 100 mM pH 8, NaCl 100mM, SDS 1% (w/v)] with 500 mg of 0.1 mm glass beads. Cells in the samples were mechanically lysed two times using MP Fastprep (Santa Ana, MP Biomedicals) for 40 s at 6 m/s. Samples were kept 5 min on ice between runs. Lysates were centrifuged 15 min at 18,000 *g* to remove beads and cell debris. DNA was extracted from the supernatant using a standard phenol/chloroform purification protocol (Thibodeau et al., 2015). Final DNA concentration was measured using the Qubit 3.0 broad range assay (Fisher Scientific, Ottawa, ON, Canada). Purified DNA samples were stored at -20°C for further analysis. The extraction was also conducted on negative controls without fecal matter.

16S rRNA gene amplicon sequencing libraries were prepared following the Illumina MiSeq protocol (Illumina, 2013). A 292 bp segment of the v4 region of the 16S rRNA gene was amplified using primers targeting the total bacterial and archaeal population (515F_Ill and 806R_Ill) (Caporaso et al., 2012). 12.5 ng of DNA was pre-amplified in a final 25 μl reaction using KAPA HiFi HotSart ReadyMix (KAPA Biosystems, Wilmington, DE, United States). The amplification was carried out for 25 cycles with a denaturation step at 95°C for 30 s, an annealing step at 55°C for 30 s, and an elongation step at 72°C for 45 s ending with a final elongation of 10 min at 72°C. Each sample was then indexed using the Nextera XT index kit (Illumina). Five microliter of the PCR product was amplified in a 20 μl reaction containing 12.5 μl of KAPA HiFi HotSart ReadyMix (KAPA Biosystems, Wilmington, DE, United States), 2.5 μl of each index primer (10 nM) and 2.5 μl of purified water. This second amplification was carried out for 8 cycles with a denaturation step at 95°C for 30 s, an annealing step at 55°C for 30 s, and an elongation step at 72°C for 30 s ending with a final elongation of 5 min at 72°C. After each PCR step, PCR product purification was conducted using Agencourt AMpure XP beads (Beckman Coulter, Brea, CA, United States). The purified PCR products were diluted to 5 nM. Fifty-one samples were pooled (5 μl of each product) and sequenced by the Illumina MiSeq sequencing system using the MiSeq Reagent Kit v2 (500 cycles).

All sequences were cleaned and analyzed using Mothur v.1.39.5 following an adapted version of the MiSeq standard operation procedure available online^1^ (accessed August 2015) (Kozich et al., 2013). First, the two complementary sets of reads were combined for each sample after removal of the primers. Sequences that were too long or that contained ambiguity were discarded. Identical sequences were grouped to reduce the necessary processing power. These unique sequences were then aligned using a Mothur adapted version of the SILVA database (silva.seed.v119).^2^ Chimeras where removed using UCHIME^3^. The sequences were clustered into operational taxonomic units (OTU) at genetic distance dissimilarity of 3% and then classified using the Mothur formatted Ribosome database project (RDP) trainset version 14.^4^ Raw reads for each sow fecal microbiota analyzed in this study are available through the NCBI SRA database under accession SRP100939.

For alpha diversity analysis, indexes were calculated in Mothur using a subsample size consisting of the lowest number of sequences in samples with 1000 iterations. The average number of OTUs, the estimation of the portion of the diversity covered by our subsampling (coverage), the diversity of the OTUs found in the samples (inverted Simpson’s index) and their evenness (Shannon evenness) was measured. Results were compared between groups using ANOVA and Student’s *t*-test and relation with parity rank using linear regression with a significance level of 0.05. For beta diversity analysis, a distance matrix comparing all the samples was created with a subsample using the same number of sequences as previously used and using Jaccard and Bray-Curtis dissimilarity index. These results were visualized using Non-metric multidimensional scaling (NMDS) graphs and the beta diversity of the different groups were statistically compared using the AMOVA test. Furthermore, OTUs that were associated with the different sow groups were identified using the Multivariate Association with Linear Models (MaAsLin) method (Morgan et al., 2012).

### Real Time Quantitative PCR of Specific Bacterial Populations

Real time PCR was conducted for specific populations on all samples: Enterobacteria (Castillo et al., 2006), Bifidobacteria (Matsuki et al., 2004) and Lactobacilli (Castillo et al., 2006) – classical indicators in microbiome analysis – as well as *Lachnospiraceae* (Wilson et al., 2014), which was identified as an interesting population because of its known role as a butyrate producer. Standard curves were built by PCR using known quantities of each targeted gene. All reactions were conducted using 10 ng of DNA using EvaGreen mastermix (Montréal Biotech, Montréal, QC, Canada) for a total reaction of 20 μl in a LightCycler 96 real time PCR (Roche diagnostics, Mannheim, Germany). Results were expressed in log of copies of the gene per 10 ng of total DNA. Levels of these populations were compared using the Student’s t-test and ANOVA (GraphPad v6, Prism, La Jolla, CA, United States).

## Results

### *Salmonella* Detection

*Salmonella* was detected in the feces of 37% (27 out of 73) of the sows that were sampled. Proportion of *Salmonella* excreting sows at the beginning of gestation (first 50 days of gestation) (21 out of 27, 78%) was significantly (Fisher’s exact, *p* < 0.05) higher than at the end of this period (last 50 days of gestation) (6 of 46, 13%). Similar results were obtained when sampling for a second time at the end of gestation the 19 sows that had already been sampled at the beginning of their gestation, with a statistically (McNemar’s, *p* < 0.05) lower proportion of positive samples at the end of the gestation (2 of 19, 11%) than at the beginning (13 of 19, 68%). Logistic regression showed no significant link between shedding and the parity of the sow (*p* > 0.05) (**Table 1**).

Of the 27 positive samples, 7 had discrepancies in the results when comparing enrichment by TBG and MSRV, with 3 samples only positive on MSRV and 4 samples only positive on TBG. All the isolates that were collected on TBG positive only samples were further tested for motility on MSRV. All isolates were motile and produced migration zones covering the whole MSRV after 24 h of incubation.

### *Salmonella* Typing

Among the isolates 3 different HRM types were detected. The most prevalent profile (HRM type 1) was present in 25 out of the 27 positive samples that were collected and was most prevalent both at the beginning and the end of gestation (19 out of 21 and 6 out of 6 respectively) (**Table 2**). For this HRM type, 10 out of the 11 isolates that were randomly selected for serotype confirmation belonged to the *S*. Infantis serotype and one was an autoagglutinating strain of the partial antigenic formula O:r:1,5. The second most frequent profile (HRM type 2) was present in 3 out of the 27 positive samples and belonged to the Derby serotype (3 isolates confirmed by serotyping) and was only detected in animals at the beginning of gestation. Finally, the third profile (HRM type 3) was present in 2 of the 27 positive samples, was only detected at the beginning of the gestation and was found to belong to the Typhimurium serotype (2 isolate confirmed by serotyping). Three samples contained isolates of two different profiles. Two samples contained *Salmonella* from profiles 1 and 2 and one sample contained *Salmonella* from profiles 1 and 3. For all the samples that contained strains of multiple profiles, all the isolates from a single BGS (TBG, MSRV near the site of inoculation and MSRV at the edge of the migration front) were of the same type. However, for these three samples, HRM type of the isolates collected from the edge of the migration zone differed from those collected near the MSRV inoculation site. The motility of these isolates was assessed. All the tested isolates were motile and covered the MSRV plate after 24 h of incubation.

**Table 2:** High resolution melt (HRM) types of *Salmonella* isolated from sows at the beginning and the end of gestation.

HRM genes profiles			HRM types	Serotype	Beginning of gestation	End of gestation
				
CRISPR 1	CRISPR 2	Yohm			(Samples)	(Samples)
1	1	1	1	Infantis/O:r:1,5	19/21	6/6
2	2	2	2	Derby	3/21	0/6
3	3	3	3	Typhimurium	2/21	0/6


### 16S rRNA Gene Amplicon Sequencing

From this section onward, samples collected from sows that were sampled once and those from sows sampled twice were analyzed together to get the highest statistical power possible. Forty-seven samples were sequenced and 45 were retained after data analysis, containing a total of 4,770,924 sequences. Most of the sequences were bacterial sequences with 4,711,793 sequences (98.8%) followed by archaeal sequences with 59,129 sequences (1.3%) and 2 unclassifiable sequences (0.00004%).

A total of 3,377 OTUs were detected, with an average of 106,020 sequences per sample. The lowest values observed for a sample were 18,037 sequences and 490 OTUs and the highest were 560,410 sequences and 1,333 OTUs.

Rarefaction was conducted and showed that after subsampling each sample at 18,037 a good coverage was obtained for all the samples (**Supplementary Figure S1**).

For the Alpha diversity, a significant although small difference in coverage was measured when comparing samples based on their *Salmonella* shedding status while no differences where found when comparing the animals based on their time of gestation. When the sows were classified both on their *Salmonella* shedding status and time of gestation, no significant variation in alpha diversity was measured. (**Table 3**) Finally, no relationship was measured between the parity number and the different alpha diversity indices (data not shown).

**Table 3:** Comparison of alpha-diversity indices across sow groups and according to *Salmonella* shedding status and time of gestation.

	Sow group				*Salmonella* status		Time of gestation	
			
	*Salmonella* Negative/Beginning	*Salmonella* Negative/End	*Salmonella* Positive/Beginning	*Salmonella* Positive/End	Positive	Negative	Beginning	End
Coverage	0.990	0.991	0.990	0.990	0.991^a^	0.990^b^	0.990	0.990
OTUs	688	637	682	676	680	642	679	647
Inverted Simpson’s	49	46	58	47	54	47	56	46
								
Shannon evenness	0.73	0.73	0.75	0.74	0.75	0.73	0.74	0.73


*The means were based on 1000 subsampling of 18,037 sequences. On the same row ^a^ is significantly different from ^b^. Significant p values of 0.05.*

**FIGURE 1:**
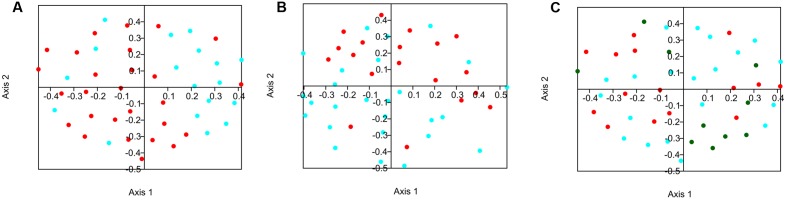
Non-metric multidimensional scaling (NMDS) plot illustrating sow fecal microbiome beta-diversity according to the time of gestation, *Salmonella* shedding status and number of gestation. **(A)** Blue = beginning of gestation; Red = end of gestation. **(B)** Red = shedding; Blue = no shedding. **(C)** Blue = low number of gestation (1–3); Green = medium number of gestation (4–5) Red = high number of gestation (6–8).

Bacterial beta-diversity was compared between groups. The similarity of the composition of communities at the OTU level was compared using the Jaccard and Bray-Curtis indexes and these distance matrices were plotted using NMDS on 2 axes when relevant (**Figures 1A–C**). Statistical variations in the composition of the microflora between the different groups were assessed using analysis of molecular variance (AMOVA) (**Table 4**). When splitting the sows into two groups, the time of gestation was revealed to have a significant effect on the beta-diversity in fecal content when using the Jaccard index. When the beta-diversity was compared based on the status of *Salmonella* shedding a significant difference was measured when using Bray-Curtis’ index. Analysis were also conducted by splitting the sows into four groups combining both their *Salmonella* shedding status and time of gestation. Significant differences were found for at least one test when comparing the group of sows shedding *Salmonella* at the beginning of gestation with the group composed of non-shedding sows at the end of gestation. Finally, the beta-diversity significantly varied in the samples collected from sows with a different number of parity when comparing sows with low (1–3) and high levels of parity (6–8) when using the Jaccard index.

**Table 4:** Beta-diversity analysis across sow groups and according to *Salmonella* shedding or time of gestation.

Compared groups		AMOVA (*p*-value)	
	
		Jaccard	Bray-Curtis
Beginning/Salmo+	Beginning/Salmo-	0.554	**0.044**
	End/Salmo+	0.014	
	End/Salmo-	**<0.001**	
Beginning/Salmo-	End/Salmo+	0.162	0.018
	End/Salmo-	0.047	
End/Salmo+	End/Salmo-	0.289	
Beginning	End	**<0.001**	0.077
Salmo+	Salmo-	0.05	**0.012**
Low parity (1–3)	High parity (6–8)	**0.012**	0.141
Low parity (1–3)	Medium parity (4–5)	0.071	
High parity (6–8)	Medium parity (4–5)	0.191	


*Based on a subsample of 18,037 sequences, there is a significant *p-*value of 0.0083 with Bonferroni correction when comparing 4 groups, 0.0166 when comparing three groups and 0.05 when comparing 2 groups. *P-*values under significant levels are in bold.*

**FIGURE 2:**
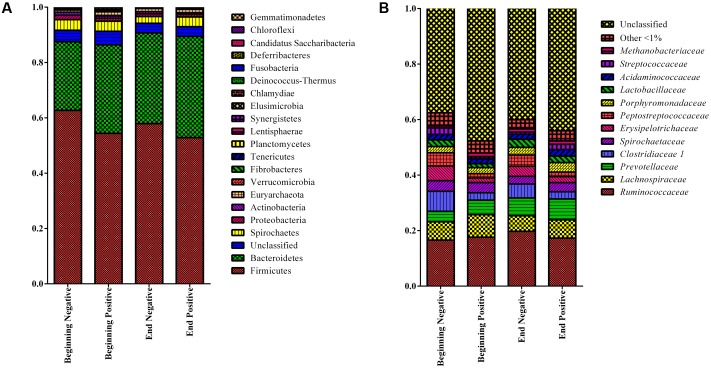
Stacked bar graphs showing the relative abundance at the phylum **(A)** and family level **(B)** levels using RDP classification (only taxa representing more than 1% of the sequences) for the four groups of sows. Beginning: Sows in the 50 first days of gestation, End: Sows in the 50 last days of gestation, Negative: Sows not shedding *Salmonella*, Positive: Sows shedding *Salmonella.*

Stacked bar graphs of the taxa found at the phylum level showed that for all the groups, the *Firmicutes* represented 56.9% of the sequences in the samples followed by the *Bacteroidetes* at 31.5% while 3.5% of the sequences could not be classified at the phylum level (**Figure 2A**). For the RDP family level taxa (taxon level 5), 12 taxa were classified and present at a level of more than 1% of the sequences and represented 53.6% of the total sequences in all the groups while 42.3% of the sequences could not be classified at the family level (**Figure 2B**).

Variation in individual clades was assessed using MaAsLin with status of shedding (positive or negative), time of gestation (beginning or end) and parity number (1–8) as metadata. For the parity, 13 OTUs classified to 10 taxa were found to be negatively associated with this value, while 13 OTUs classified to 5 taxa were positively associated. For the *Salmonella* shedding status, 11 OTUs classified to 6 taxa were positively associated with shedding animals, while 3 OTUs classified to 2 taxa were associated with non-shedding animal. Finally, for the gestation time, 58 OTUs classified to 22 taxa were positively associated with animals at the beginning of the gestation, while 20 OTUs classified to 10 taxa were positively associated with animals at the end of the gestation (**Supplementary Data S1**).

### Real Time Quantitative PCR for Specific Populations

**FIGURE 3:**
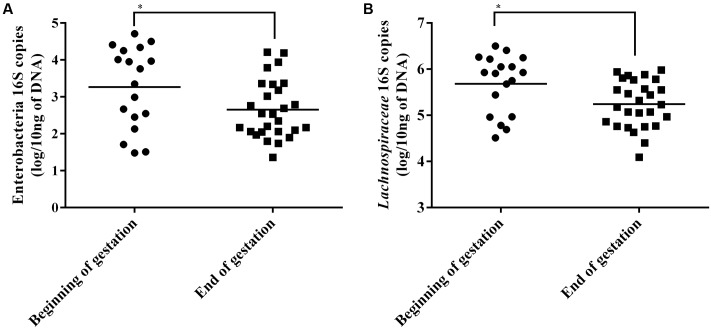
Enterobacteria **(A)** and *Lachnospiraceae* **(B)** 16S rRNA gene copies in sow fecal content at beginning and end of gestation. Horizontal bars represent the mean of each group, each point represent the fecal content of a single sow. ^∗^Indicates *p* < 0.05.

A difference between the sows at the beginning of gestation and the end of gestation was detected for the Enterobacteria group with a mean of 0.61 log of copies more at the beginning of gestation (Student’s *t*-test, *p* < 0.05) (**Figure 3A**). Furthermore, a significant difference of 0.44 log more copies at the beginning of gestation than at the end was detected for the *Lachnospiraceae* family (**Figure 3B**). No differences were detected for the Lactobacilli or Bifidobacteria quantifications. When comparing the sows by *Salmonella* status or in groups that combine both shedding status and time of gestation, no significant differences were measured for the four tested populations.

## Discussion

In this study, we investigated the dynamics of shedding of *Salmonella* by sows in terms of variation of both shedding prevalence and type of strains excreted during gestation in an industrial setting in Quebec. We also evaluated the modification of the fecal microbiota during gestation and the possible link with the shedding of *Salmonella*.

High percentages of *Salmonella* shedding animals were detected on the farm with a total of 37% of sampled sows positive for the bacteria. These results are similar to what had already been measured in other provinces of Canada, with 38% of the individual samples found to be positive in Saskatchewan and Alberta (Wilkins et al., 2010). However, Wilkins’ study used samples of 10 g of feces and since it is known that fecal sample weight affects sensitivity, the real prevalence could have been underestimated (Funk et al., 2000; Wilkins et al., 2010). Our results confirm that sows are reservoirs of *Salmonella*.

When separating sows at the beginning and the end of gestation, we measured a significant variation of shedding, with higher number of animals shedding (78%) at the beginning when compared to the end (13 %). Description of variations of *Salmonella* shedding during the production cycle of sows is scarce. Some research has been done in an Italian study conducted in a farrow-to-finish farm that showed a low shedding of 0.6% in late gestation (2 weeks before parturition) and a high shedding of 26.5% in post-weaning (1 week after parturition) (Magistrali et al., 2011). Similar results were also found in Belgian herds were the prevalence of *Salmonella* shedding by sows was less than 10% during late gestation, around farrowing and during lactation and raised significantly 7 days after weaning (Nollet et al., 2005b). Hence it seems that the number of sows shedding *Salmonella* is at its highest when the sows are not in contact with the piglets.

The majority of positive samples (25 of the 27) from this farm contained isolates of HRM type that belong to the Infantis serovar. The isolates from this serovar could represent a major resident strain present on the farm. A possible association between this serotype and the maternity phase of the production has been described in Canadian farrow-to-finish farms (Wilkins et al., 2010). However, this serotype was not one of the 5 most frequently found in the samples submitted to the Public Health Agency of Canada for antimicrobial susceptibility testing, showing that this serotype is somewhat rare in human cases in Canada (Public Health Agency of Canada [PHAC], 2016). Also, detected at lower levels, were isolates of two other HRM types that belong to strains of the Derby and Typhimurium serovars. The low diversity of serotypes in the farm is similar to what was found in other studies in maternity and fattening units. In studies conducted in Europe, similar strains were found at different stages of production in farrow to finish farms (Nollet et al., 2005a), however, in studies carried out on multi-site production systems, like in the present study, the link between strains found in maternity and fattening units has not been clearly established. In these production systems, changes in the major strains present at different stages of production and appearance of new serotypes at the nursery and fattening stages were detected (Funk et al., 2001). This lack of a detected link in this type of production could be the result of the low sensitivity of the method to detect minor strains in samples containing many strains.

In this study, to get the highest sensitivity and the best snapshot of the different serotypes that were excreted by the sows, a modified *Salmonella* detection method was used. Since it is known that different serovars of *Salmonella* have different growth rates in selective enrichment media, using multiple media could help get better representativeness of the excreted strains by the sows (Gorski, 2012; De Busser et al., 2013). For seven samples, only one of the two selective enrichment media was positive. For four of these samples, only the TBG was positive. Since *Salmonella* detection on MSRV is based on the motility of the bacteria, these results could show that these isolates are non-motile. The motility of the isolates that were not detected on MSRV was tested, and all the isolates formed migration zones on the media covering the whole surface. Therefore, the lack of detection could not be explained by strains that are non-motile. It is possible that the lack of detection on either TBG or MRSV could be a result of the competitive flora present in the media after the first enrichment in BPW. The method used in this study also proved to be useful in three samples where strains of different serotypes were recovered at different distances of migration on the MSRV media. Interestingly, in two of these samples, because the isolate recovered from the MSRV at the inoculation site was different from those recovered on TBG and at the edge of the migration zone on MRSV, using the standard method would have resulted in a loss of information. The motility of these strains was also tested as previously described and with identical results. These results confirm the importance of using multiple enrichment methods and possibly multiple migration distances on MSRV when trying to get an accurate account of the different strains of *Salmonella* present on a farm.

In this study, to explain the mechanisms that are at play in the variation of shedding between the beginning and end of gestation, the microbiota of sows was studied. We described for the first time the beta diversity of the fecal microbiota of *Salmonella* excreting and non-excreting sows at the beginning and end of gestation.

The AMOVA statistical test showed that the gestation time, the *Salmonella* shedding status and the parity number significantly affected the populations found in the fecal flora. However, the gestation time and parity number variations were only significant using the Jaccard index which only considers the presence or absence of the OTUs while the status of *Salmonella* shedding variations were only significant when using the Bray-Curtis index which also considers the number of time the OTUs are found in the samples (Legendre and Legendre, 2012). This shows that while these factors all have significant impact on the flora they have different impacts on the microflora composition. Variation in the microbiota during gestation has already been described in other studies, mostly in human, with non-convergent conclusions. Two studies showed significant differences between the beginning and end of gestation in humans (first to third trimester) (Collado et al., 2008; Koren et al., 2012). However, another study showed no significant variation in the microbiota during gestation (DiGiulio et al., 2015). Similarly, *Salmonella* has been shown to be associated with variations in the flora of both challenged and naturally infected animals (Bearson et al., 2013; Borewicz et al., 2015). However, it is the first time that these modifications are measured in sows.

MaAsLin was used to identify biomarkers linked with the time of gestation, status of excretion and number of parity while considering confounding factors. Three OTUs negatively associated with the number of parity were classified to the genus level to *Succinivibrio* (1 OTU), *Coprococcus* (1 OTU), *Blautia* (1 OTU). While only one OTU positively associated with the number of parity was classified to the genus level at 63% to the *Clostridium* cluster IV. Most of the statistically significant taxa were associated with the time of gestation. Five OTUs that were positively associated with the end of the gestation, when *Salmonella* shedding is at it’s lowest, were classified at the genus level to *Alloprevotella* (2 OTUs), *Prevotella* (2 OTUs) and *Clostridium* cluster IV (1 OTU). All those genera are known short chain fatty acid producers and could affect the virulence of *Salmonella*. Indeed, it has been shown that butyrate, regulates the expression of SPI-1, reducing the invasiveness of *Salmonella* (Gantois et al., 2006). However, supplementing finishing pigs with butyric acid was tested with variable level of success. A study showing significant reduction of *Salmonella* levels in feces after sodium butyrate supplementation (Lynch et al., 2017) while another showed no effect in reducing *Salmonella* carriage under farm conditions (Walia et al., 2016). Ten OTUs that were negatively associated with the time of gestation, hence higher at the beginning of the gestation when *Salmonella* shedding is at its highest, were classified at the genus level. Most interestingly *Bacteroides* (1 OTU), *Blautia* (1 OTU), *Coprococcus* (1 OTU) and *Ruminococcus* (OTU 1) which are all known short chain fatty acid producers (Pryde et al., 2002; den Besten et al., 2013; Park et al., 2013; Rivière et al., 2016). Another study already showed a reduction of health-associated species such as butyrate producers, like *Coprococcus* and *Ruminoccocus* in the current study, in the last trimester of human gestation, coupled with an augmentation of the Proteobacteria which is frequently found in inflammation-associated dysbiosis (Koren et al., 2012). These results may seem unexpected in the context of the higher level of shedding of *Salmonella* at the beginning of the gestation, since as previously mentioned short chain fatty acids, such as butyric acid, are known to reduce *Salmonella*’s virulence and invasiveness. However, the *Bacteroides* and *Blautia* genus are acetate producers. This short chain fatty acid has been shown *in vitro* to activate virulence genes in *Salmonella* (Lawhon et al., 2002). Hence, the higher presence of the genus at the beginning of gestation could be linked with the higher excretion. The only significantly varying archaeal taxon in the study, the genus *Methanomassiliicoccus*, which is a methane producing microorganism, was also associated with the beginning of the gestation. These results show that the reduction of *Salmonella* shedding at the end of the gestation could be linked with specific taxa of short-chain fatty acid producers or that other factors such as hormonal and immunological changes are also in play. No OTUs positively associated with the excretion of *Salmonella* could be classified at the genus level. However, one interesting OTU was classified to the *Enterobacteriaceae* family. This result is coherent with the higher number of excreting animals and show that the conditions that are favorable for *Salmonella* shedding could also be favorable for other *Enterobacteriaceae*. Only one OTU associated with animals not shedding *Salmonella* was classified to the genus level to the *Clostridium* cluster XI. More research will be necessary to confirm causal relationships between the abovementioned taxa and *Salmonella* shedding.

We observed a higher abundance of Enterobacteria and of *Lachnospiraceae* in sows at the beginning of the gestation using qPCR. The Enterobacteria results are consistent with our results showing that this time point was also associated with a higher level of *Salmonella* shedding but also with the association between shedding animals and the *Enterobacteriaceae* family found with MaAsLin. However, since *Salmonella* is only found in low levels compared to other Enterobacteria this significant reduction must be the results of the reduction of multiple genus contained in this group. The *Lachnospiraceae* results also seem to be consistent with the sequencing results which showed that 3 OTUs classified to the *Lachnospiraceae* family and 1 *Coprococcus* OTU which is part of this family were enriched at the beginning of the gestation.

The cause and effect of these changes in the microbiota during gestation and *Salmonella* shedding are not well known. However, similarly to the important change in the microbiota between the beginning and the end of gestation observed here, it has been shown that the fecal microbiota varies greatly between the first and the third semester in pregnant women (Collado et al., 2008; Koren et al., 2012). These changes were shown to contribute to modification in the metabolism and immunity of the host, playing a role, for example, in weight gain associated with pregnancy (Nuriel-Ohayon et al., 2016). Similar modifications could be at play during sow gestation, resulting in lower *Salmonella* carriage later in gestation. As for the mechanisms that are responsible for these changes, it has been hypothesized that changes in the mucosal immune system or at the hormonal level could be responsible for the variation of certain bacterial populations (Koren et al., 2012). For the changes associated with *Salmonella* shedding, another studies have found that a natural infection by this bacteria can alter the microflora in swine (Borewicz et al., 2015). It is known that *Salmonella* possess multiple strategies to compete with the intestinal microflora (Thiennimitr et al., 2012). However, in swine, the mechanisms that are in cause are still not well known. Since *Salmonella* infection in swine is mostly asymptomatic, authors have hypothesized that in swine mechanisms other than inflammation could be responsible for this variation (Kim and Isaacson, 2017).

## Conclusion

In the present study, we showed that gestating sows are a possible reservoir of *Salmonella* with high levels of contamination and that standard detection methods are not sensitive enough to describe the entire diversity of the *Salmonella* strains present on the farm. We also showed that the level of shedding was variable during gestation with significantly higher shedding at the beginning rather than at the end of gestation. We also observed for the first time a significant change in the microbiota during sow gestation and identified interesting taxa such as *Alloprevotella*, *Prevotella* and the cluster IV *Clostridium*, which could be linked to the reduction of *Salmonella* shedding.

## Author Contributions

GL-G designed the experiments, did all experimentations, analyzed all results, discussed the results and wrote the manuscript. AL designed the experiments, discussed the results and revised the manuscript. ÉY designed the experiments, discussed the results and revised the manuscript. AT did some experimentations, discussed the results and revised the manuscript. PF designed the experiments, discussed the results and revised the manuscript.

## Conflict of Interest Statement

The authors declare that the research was conducted in the absence of any commercial or financial relationships that could be construed as a potential conflict of interest.

## Footnotes

1 https://www.mothur.org/wiki/MiSeq_SOP

2 https://www.mothur.org/wiki/Silva_reference_files

3 http://drive5.com/usearch

4 https://www.mothur.org/wiki/RDP_reference_files

## Supplementary Material

**DATA S1 |** Complete list of features associated with gestation time, parity number and *Salmonella* shedding status.

**FIGURE S1 |** Rarefaction curve comparing the number reads with the average number of OTUs found in all sequenced samples of sows’ feces for 1000 iterations.
