# Supplementary figures and images for: Reduction of Salmonella Shedding by Sows during Gestation in Relation to Its Fecal Microbiome
Source: Front Microbiol. 2017 Nov 10;8:2219. doi: 10.3389/fmicb.2017.02219 (PMC5701629; doi:10.3389/fmicb.2017.02219)

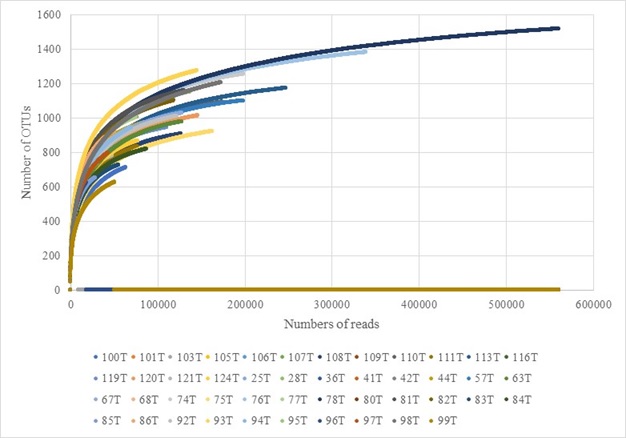

Supplement: Supplementary file 2 [file Image_1.JPEG]
